# The role of resistance exercise training for improving cardiorespiratory fitness in healthy older adults: a systematic review and meta-analysis

**DOI:** 10.1093/ageing/afac143

**Published:** 2022-06-21

**Authors:** Thomas F F Smart, Brett Doleman, Jacob Hatt, Melanie Paul, Suzanne Toft, Jonathan N Lund, Bethan E Phillips

**Affiliations:** Centre of Metabolism, Ageing and Physiology (COMAP), School of Medicine, Royal Derby Hospital Centre, University of Nottingham, Derby DE22 3DT, UK; MRC-Versus Arthritis Centre for Musculoskeletal Ageing Research and Nottingham National Institute for Health Research (NIHR) Biomedical Research Centre (BRC), Derby, UK; Department of Surgery and Anaesthetics, Royal Derby Hospital, Derby DE22 3NE, UK; Centre of Metabolism, Ageing and Physiology (COMAP), School of Medicine, Royal Derby Hospital Centre, University of Nottingham, Derby DE22 3DT, UK; MRC-Versus Arthritis Centre for Musculoskeletal Ageing Research and Nottingham National Institute for Health Research (NIHR) Biomedical Research Centre (BRC), Derby, UK; Department of Surgery and Anaesthetics, Royal Derby Hospital, Derby DE22 3NE, UK; Centre of Metabolism, Ageing and Physiology (COMAP), School of Medicine, Royal Derby Hospital Centre, University of Nottingham, Derby DE22 3DT, UK; MRC-Versus Arthritis Centre for Musculoskeletal Ageing Research and Nottingham National Institute for Health Research (NIHR) Biomedical Research Centre (BRC), Derby, UK; Department of Surgery and Anaesthetics, Royal Derby Hospital, Derby DE22 3NE, UK; Centre of Metabolism, Ageing and Physiology (COMAP), School of Medicine, Royal Derby Hospital Centre, University of Nottingham, Derby DE22 3DT, UK; MRC-Versus Arthritis Centre for Musculoskeletal Ageing Research and Nottingham National Institute for Health Research (NIHR) Biomedical Research Centre (BRC), Derby, UK; Department of Surgery and Anaesthetics, Royal Derby Hospital, Derby DE22 3NE, UK; Department of Surgery and Anaesthetics, Royal Derby Hospital, Derby DE22 3NE, UK; Centre of Metabolism, Ageing and Physiology (COMAP), School of Medicine, Royal Derby Hospital Centre, University of Nottingham, Derby DE22 3DT, UK; MRC-Versus Arthritis Centre for Musculoskeletal Ageing Research and Nottingham National Institute for Health Research (NIHR) Biomedical Research Centre (BRC), Derby, UK; Department of Surgery and Anaesthetics, Royal Derby Hospital, Derby DE22 3NE, UK; Centre of Metabolism, Ageing and Physiology (COMAP), School of Medicine, Royal Derby Hospital Centre, University of Nottingham, Derby DE22 3DT, UK; MRC-Versus Arthritis Centre for Musculoskeletal Ageing Research and Nottingham National Institute for Health Research (NIHR) Biomedical Research Centre (BRC), Derby, UK

**Keywords:** ageing, resistance exercise, cardiorespiratory fitness, health, older people, systematic review

## Abstract

**Background:**

Declines in cardiorespiratory fitness (CRF) and muscle mass are both associated with advancing age and each of these declines is associated with worse health outcomes. Resistance exercise training (RET) has previously been shown to improve muscle mass and function in the older population. If RET is also able to improve CRF, as it has been shown to do in younger populations, it has the potential to improve multiple health outcomes in the expanding older population.

**Methods:**

This systematic review aimed to identify the role of RET for improving CRF in healthy older adults. A search across CINAHL, MEDLINE, EMBASE and EMCARE databases was conducted with meta-analysis performed on eligible papers to identify improvements in established CRF parameters (VO_2_ peak, aerobic threshold (AT), 6-minute walking distance test (6MWT) following RET intervention. Main eligibility criteria included older adults (aged over 60), healthy cohorts (disease-specific cohorts were excluded) and RET intervention.

**Results:**

Thirty-seven eligible studies were identified. Meta-analysis revealed a significant improvement in VO_2_ peak (MD 1.89 ml/kg/min; 95% confidence interval (CI) 1.21–2.57 ml/kg/min), AT (MD 1.27 ml/kg/min; 95% CI 0.44–2.09 ml/kg/min) and 6MWT (MD 30.89; 95% CI 26.7–35.08) in RET interventions less than 24 weeks. There was no difference in VO_2_ peak or 6MWT in interventions longer than 24 weeks.

**Discussion:**

This systematic review adds to a growing body of evidence supporting the implementation of RET in the older population for improving whole-body health, particularly in time-limited timeframes.

## Key Points

- Resistance exercise training improves both cardiorespiratory fitness and muscle strength in older adults.
- Resistance exercise training improves multiple measures of cardiorespiratory fitness within a 24-week intervention in older adult.
- Resistance exercise training could provide improvement to whole body health within time-limited clinical pathways.

## Introduction

Decades of increasing life expectancy have resulted in those over the age of 65 years becoming an increasing proportion of the UK population [1], with this trend replicated across much of the world. However, despite this increase in life span, there has not been an equal match in the rise of healthy life years lived. This disparity means that in the UK an estimated 20% of life is spent in poor health [2]. With predictions that 7% of the UK population will be over the age of 85 years by 2066 [3], it is crucial to develop evidence-based interventions, which can improve the proportion of life spent independent and healthy.

Cardiorespiratory fitness (CRF) is an important aspect of health and is known to decline in a non-linear manner as part of the ageing process [4–6]. Lower levels of CRF are associated with reduced life expectancy, increased healthcare costs and worse clinical outcomes [7–9]. The decline in CRF with advancing age is paralleled by a decrease in skeletal muscle mass and function, particularly muscle strength. This age-associated decline in muscle mass and function is termed sarcopenia [10, 11]. As with reduced CRF, sarcopenia has been linked with shorter life expectancy and is an independent predictor of poor clinical (i.e. surgical) outcomes [12–15]. Both low CRF and sarcopenia are viewed to be key components of frailty [16].

Exercise training-induced changes in CRF are thought to be due to the release and action of myokines [17–19]. Myokines induce changes locally in the muscle by regulating muscle development and enhancing muscle function. Myokines have distal effects, largely on adipose stores, enhancing metabolic pathways and inhibiting inflammatory responses [20].

Traditionally, aerobic exercise training (AET) has been used to improve CRF (including in older adults) [21, 22]; however, this form of training fails to improve muscle mass [23]. Conversely, resistance exercise training (RET) is the most employed modality to improve both muscle mass and function [24]. In young adults, RET has also been shown to improve CRF [25]; however, the impact of RET on the CRF of older adults is not well established. Building on observations in younger adults, this systematic review aims to explore the impact of RET on the CRF of healthy older adults.

## Methods

The systematic review was registered on PROSPERO (ID CRD42020223356). A literature search was performed across CINAHL, MEDLINE, EMBASE, EMCARE, PubMed and Cochrane databases using a PICO protocol (Appendix 1). Databases were searched from database creation to 21 January 22. Only randomised control studies available in the English language were deemed eligible for inclusion.

### Population

The population was determined to be healthy older adults (male or female, aged over 60 years), Given the high prevalence of chronic diseases within the older population, studies were included if they included a participant with chronic illnesses; however, studies were excluded if participants were recruited according to a disease profile (e.g. a group of participants all suffering from chronic obstructive pulmonary disease or heart failure would be excluded).

### Intervention

The intervention was defined as strength or resistance-based exercise training involving multiple training sessions. Studies were excluded if the intervention provided combined exercise training (involving both aerobic and RET), or if assessment was conducted after a single training session (i.e. no training program was delivered). Studies with exercise programs longer than 24 weeks were included in separate sub-analysis to improve homogeneity between short-term and long-term studies. A control group was defined as a group performing no exercise or a sham exercise intervention.

### Paper selection process

**Figure 1:**
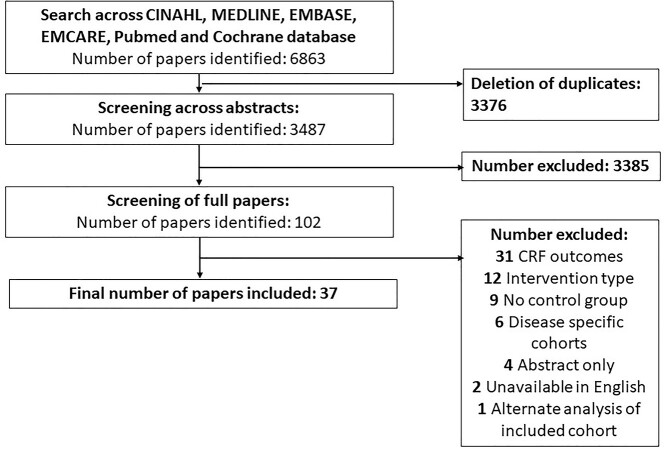
Paper identification via PRISMA Guidelines.

Two authors (M.P. and T.S.) independently screened abstracts using RAYYAN [26], full papers were then screened and any disagreements were resolved by consensus. A flow diagram of study identification can be seen in Figure 1.

### Outcome measures

The primary outcome measures of CRF included were maximal oxygen consumption (VO_2_ Peak), 6-minute walking test (6MWT) distance and anaerobic threshold (AT).

### Statistical analysis

Effect estimates are reported as mean differences (MD) with 95% confidence intervals (CIs). Due to the clinical heterogeneity between shorter (<24 weeks) and longer term (>24 weeks) interventions, these were analysed separately. Due to inconsistent reporting of mean changes and change standard deviations (SDs), we calculated these using formulae from the Cochrane Handbook. We assumed a correlation coefficient of 0.7 between baseline and final values based on analysis of our previous, similar data [27]. When data were reported using l/min, we transformed data to ml/kg/min using average weight values from the study. We performed meta-analysis using a restricted maximum likelihood random effects model [28]. When more than 10 studies were included in a meta-analysis, we produced contour-enhanced funnel plots (*P* = 0.05 contour) and tested for possible publication bias using Egger’s linear regression test (*P* < 0.1). We used the *I*_2_ statistic to assess statistical heterogeneity. We also investigated heterogeneity by number of weeks of the intervention using a restricted maximum likelihood random effects meta-regression with Knapp–Hartung modification (>10 included studies with *P* < 0.1). This is reported with the *R*_2_ analogue. We used GRADE to assess the certainty of evidence for each outcome. We conducted sensitivity analysis by assuming different correlation coefficients when calculating change SDs (0.5 and 0.9). All data were extracted into Stata Version 16 for analysis.

## Results

### Included studies

Thirty-seven studies were identified as eligible. Study basic information and intervention design can be seen in Appendix 2A. Study demographics can be seen in Appendix 2B.

### Risk of bias

All papers were analysed for risk of bias using the Cochrane risk-of-bias tool for randomised control trials (RoB2) [29]. Details of this analysis are presented in Table 1.

**Table 1:** Risk of bias analysis using RoB2

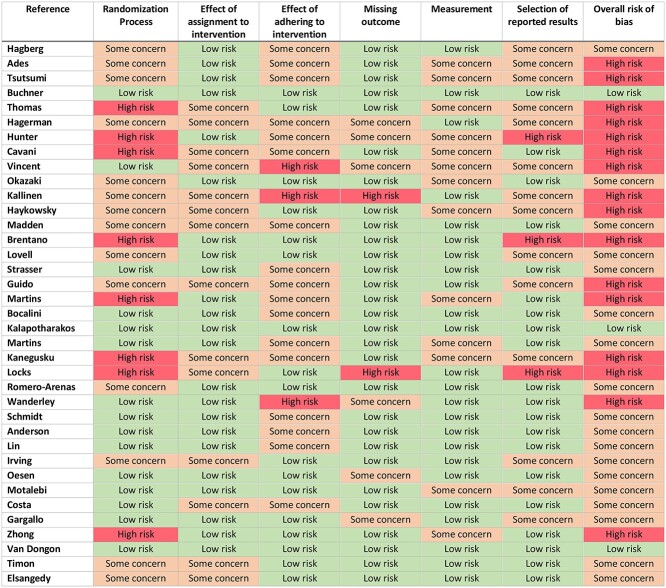

### VO_2_ peak

**Figure 2:**
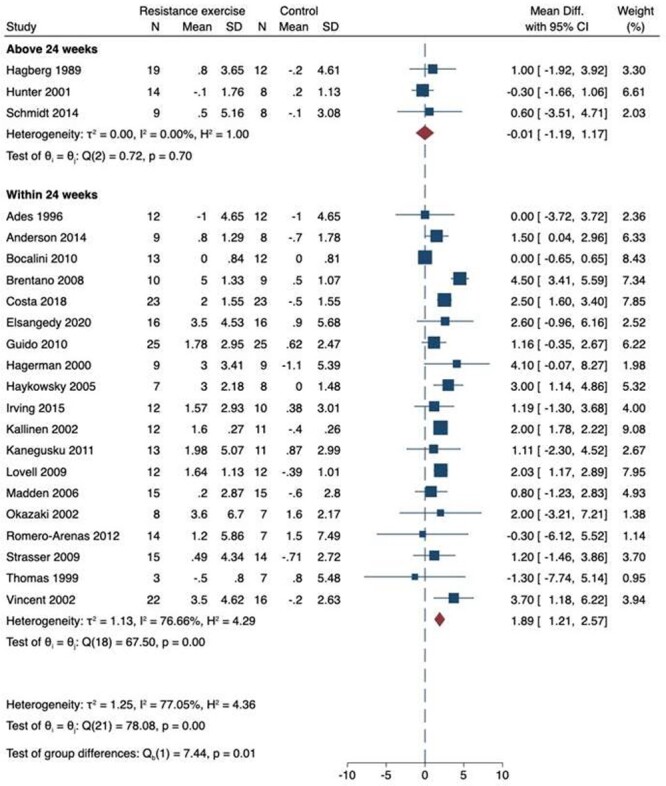
Meta-analysis of 22 studies exploring the effect of resistance exercise training on VO_2_ peak in healthy older adults.

We included 24 studies for VO_2_ peak; of these, 22 studies were eligible for meta-analysis as shown in Figure 2 [30–51]. Three of these studies had intervention periods over 24 weeks [32, 39, 43]. Overall, there was a significant increase in VO_2_ peak with RET if the intervention was 24 weeks or less (MD 1.89 ml/kg/min; 95% CI 1.21–2.57 ml/kg/min). There was no difference in VO_2_ peak with RET intervention periods longer than 24 weeks (MD −0.01 ml/kg/min; 95% CI −1.19 to 1.17 ml/kg/min). There was no funnel plot asymmetry on visual inspection and there was no evidence of possible publication bias on statistical testing (*P* = 0.54) (Appendix 3A). There was considerable statistical heterogeneity when the intervention was 24 weeks or less (*I*_2_ = 77%), but not when the intervention was longer than 24 weeks (*I*_2_ = 0%). On meta-regression analysis, the improvement in VO_2_ peak was not predicted by number of weeks of the intervention (*R*_2_ = 2%; *P* = 0.42). Similar estimates were obtained on sensitivity analysis. The certainty of evidence was moderate to high due to no evidence of publication bias and narrow confidence intervals with a large sample size, however given the risk of bias across the papers some concerns remained.

Two papers reported VO_2_ outcomes as percentage change and were therefore not included in analysis. One paper reported no significant change when compared to control with a 24- to 26-week intervention (0.3% versus 0.3%) [41]. The other paper reported that there was a change between 0.2 and 2.9 ml/kg/min across all groups when comparing 12 weeks of high-intensity RET, low-intensity RET or control [52].

### Six-minute walk test

**Figure 3:**
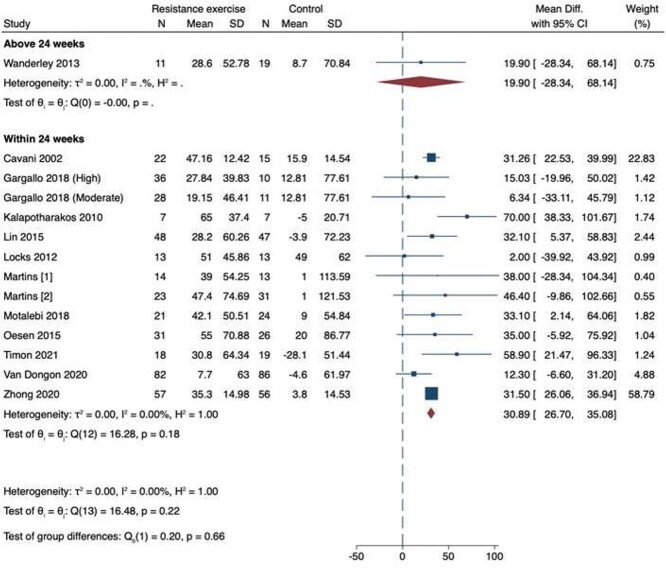
Meta-analysis of 13 papers exploring the effect of resistance exercise training on 6MWT in healthy older adults.

We included 13 studies for 6MWT as shown in Figure 3 [53–65]. One study had an intervention period above 24 weeks and 12 studies 24 weeks and below. There was an increase in 6MWT in the shorter (24 weeks and less) (MD 30.89; 95% CI 26.7–35.08) but not the longer (more than 24 weeks) (MD 19.9; 95% CI −28.34 to 68.14) studies. There was no evidence of statistical heterogeneity for either duration of intervention (*I*_2_ = 0%). There was no funnel plot asymmetry on visual inspection and there was no evidence of possible publication bias on statistical testing (*P* = 0.91) (Appendix 3B). On meta-regression, length of intervention did not predict increase in 6MWT (*R*_2_ = 27%; *P* = 0.81). Similar estimates were obtained on sensitivity analysis. The certainty of evidence was moderate for interventions less than 24 weeks given the number of studies included with no evidence of publication bias, however with high weighting on a single study with a high risk of bias concerns remain.

### Aerobic threshold

**Figure 4:**
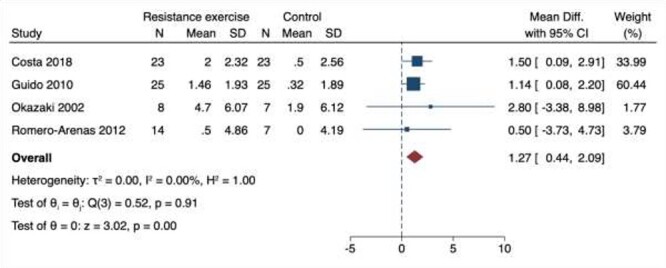
Meta-analysis of four papers exploring the effect of resistance exercise training on AT in healthy older adults.

We included four studies for AT as shown in Figure 4 [37, 38, 48, 49]. All interventions were 24 weeks or less. Overall, there was a significant increase in AT with RET (MD 1.27 ml/kg/min; 95% CI 0.44–2.09 ml/kg/min). There was no evidence of statistical heterogeneity (*I*_2_ = 0%). There were too few studies to assess publication bias or conduct meta-regression. Similar estimates were obtained on sensitivity analysis. The certainty of evidence was very low, given the low number of papers included and high weighting to a single study.

## Discussion

Given the known benefits of RET on muscle mass and strength, but limited knowledge of its impact upon CRF in older adults, we sought to systematically review and quantify the impacts of RET on CRF specifically in this age group. The main finding is that the CRF of older adults can be improved by a short-term RET program. Curiously, this improvement is not apparent with longer term RET.

This review includes a total of 1,641 older adults undergoing RET (832) or a no/sham-intervention control (809). The average age of those completing RET was 70.21, with the control participants aged 70.54. The dropout rate of participants across all studies was low at 10.2%, suggesting that RET is well tolerated by older adults. This dropout rate is comparable to the 12% previously reported for AET in older adults [66]. In this review, the most cited reasons for non-completion were non-clinical, including (i) geographical relocation, (ii) time commitment and (iii) failure to attend for reassessment. There was a low reported incidence of clinical reasons for dropout. Those that were reported included undergoing surgery unrelated to the study, or minor musculoskeletal injury. Kallinen *et al*. reported one death due to a myocardial infarction and another participant developing unstable angina [51]. Both cardiovascular events were reported to have started >2 days after a RET session and as such were not felt to be directly caused by the training. It should however be noted that in retrospect, an area of ischaemic change was identified on exercise ECG in the participant who developed unstable angina. Given that all modalities of exercise training carry cardiovascular risk in previously untrained individuals, and in accordance with guidelines published by the American College of Sports Medicine, all individuals commencing exercise training for the first time or after a prolonged hiatus are recommended to have a medical screening [67].

Despite the potential positive impact of RET for improving CRF in older adults highlighted by this review, there is difficulty in performing direct comparisons across studies given the wide variety of RET provided. When considering the FITT (frequency, intensity, time and type) principles of exercise training [67], length of training is only one important variable for exercise adaptation and training progression. Another core concept in exercise training is that of ‘overload’; the concept of a new challenge (often by altering FITT components) being required for progression [68]. The studies included in this review involved a variety of techniques to both set and progress the RET. These techniques included using a progressive rise in resistance according to the Borg Rating of Perceived Exertion scale [69], and performing one repetition maximum reassessments throughout the RET program [70]; with not all studies stipulating how the resistance was set or adjusted. There was also variety in the type of RET used with machine weights, free weights and alternatives (such as resistance bands) used. It is therefore perhaps even more promising that, despite this significant variability across studies, RET was still deemed able to improve the CRF of older adults within a short-term exercise program.

Another difficulty allowing for direct comparison is the different methods used to ascertain the measures. For example, in the most reported outcome, VO_2_ peak, both treadmills and cycle ergometers were used. This is with known differences in the O_2_ cost of these exercise modalities [71], and reported differences in maximum O_2_ uptake between them [72].

The improvement in CRF following a short-term RET programme is arguably of great importance when promoting exercise for older adults within clinical pathways. Improvement in CRF, combined with the established benefits of muscle mass and strength gains with RET [24], means that RET has the potential to impact on multiple facets of systems physiology known to decline with advancing age. RET could disrupt multiple processes of the frailty model and thus promote whole-body health. This proposition is supported by work demonstrating the impact of RET on functional outcomes such as reduced likelihood of falls [73, 74].

That all three of our validated measures of CRF (VO_2_, 6MWT and AT) showed improvements following short-term RET, also suggests that in a clinical or resource-limited setting, ‘bedside’ assessments of CRF such as 6MWT may be able to determine the benefits of RET, without the need for labour (personnel and equipment) intensive cardiopulmonary exercise testing. However, that longer RET did not seem to elicit improvements in CRF is curious. Beyond the small (*n* = 4) number of studies which included longer duration interventions [32, 43, 58, 75], one possible reason for this finding is that 50% of these studies employed an intervention, which was only two sessions per week, compared to three sessions each week being the most reported frequency in the shorter interventions. In addition, it may be speculated that individuals engaged in a regular RET intervention over a longer period may reduce their other activity outside of these sessions, although habitual physical activity was seldom reported in the studies included in this review.

In conclusion, this systematic review demonstrates that short-term RET improves CRF in healthy older adults, based on evidence using multiple measures of this health-related parameter. This finding, combined with the already established evidence base that RET improves muscle mass and function [24], suggests that RET should be an integral aspect of exercise promotion for an ageing population. Further, that ‘lack of time’ is a commonly cited barrier to exercise in older adults [76] and that older adults commonly face time-limited clinical pathways where improvements in physiological resilience have been shown to be beneficial (i.e. surgical prehabilitation [77]), RET may be able to elicit benefit in two key components of whole body health (i.e. muscle and CRF) in older adults.

## Supplementary Material

aa-21-1874-File003_afac143
