# Supplementary material for: The role of resistance exercise training for improving cardiorespiratory fitness in healthy older adults: a systematic review and meta-analysis
Source: Age Ageing. 2022 Jun 21;51(6):afac143. doi: 10.1093/ageing/afac143 (PMC9220026; doi:10.1093/ageing/afac143)
Supplement: aa-21-1874-File003_afac143 [file aa-21-1874-file003_afac143.docx]

The role of resistance exercise training for improving cardiorespiratory fitness in healthy older adults: A systematic review and meta-analysis

**SUPPLEMENTARY DATA**

- **Appendix 1:** Search strategy including search terms used
- **Appendix 2A:** Study information, including study location, length, RET design, control design
- **Appendix 2B:** Study demographics (Mean (S.D.))
- **Appendix 3A:** Contour-enhanced funnel plots of VO_2_ peak (p=0.54)
- **Appendix 3B:** Contour-enhanced funnel plots of 6MWT (p=0.91)

**Appendix 1: Search strategy including search terms used**

(older adult* OR old* OR elder* OR senior* OR “late life” OR “later life”).ti,ab OR aged.ti OR exp AGED/

AND

(health* OR healthy ag*ing OR “physical function” and “functional fitness”).mp OR exp HEALTH/ OR "HEALTHY AGING"/ OR exp "PHYSICAL FITNESS"/ OR exp "HEALTHY LIFESTYLE"/

AND

(resistance ADJ4 training).mp OR ("resistance exercise*" OR "strength training" OR ("weight* training" OR "weight* bearing strengthening").mp OR (weight* ADJ4 lift*).mp OR exp "RESISTANCE TRAINING"/ OR exp "MUSCLE STRENGTHENING"/ OR exp "WEIGHT LIFTING"/

AND

(cardio* fitness OR “cardi* ADJ2 fitness”).mp OR (VO2peak OR oxygen ADJ2 uptake OR “heart rate” OR “6-minute walk test” or “step test”).mp OR "CARDIORESPIRATORY FITNESS"/ OR exp "EXERCISE TEST"/ OR exp "HEART FUNCTION TESTS"/ OR exp "RESPIRATORY FUNCTION TESTS"/ OR exp "OXYGEN CONSUMPTION"/ OR exp "CARDIOVASCULAR PHYSIOLOGICAL PHENOMENA"/ OR exp "VENTRICULAR FUNCTION"/ OR exp "HEART RATE"/ OR "WALK TEST"/ OR exp "BLOOD PRESSURE"/ OR "EXERCISE TOLERANCE"/

Appendix 2

| **Reference** | **Year** | **Country** | **Length of Study (Weeks)** | **Resistance Training Design** | | | | | | | **Control**  **Design** |
| --- | --- | --- | --- | --- | --- | --- | --- | --- | --- | --- | --- |
|  |  |  |  | Number of sessions per week | Resistance provided by | Number of exercises | Sets + Repetitions | Resistance setting | Resistance progression | Supervised? |  |
| **Hagberg JM** | 1989 | USA | 26 | 3 | Resistance machines | 12 | 1 set 10 reps | Unspecified | On completion of 12 reps | Yes | No training |
| **Ades** | 1996 | USA | 12 | 3 | Resistance machines | 7 | 3 sets 8 reps | Started 50%, increased to 90% of 1RM | "intermittently" | Unspecified | No training |
| **Tsutsumi T (High intensity)** | 1997 | USA | 12 | 3 | Resistance machines | 12 | 8-12 reps (unspecified set) | 75-85% 1RM | Every 4-6 sessions | Yes | No training |
| **Tsutsumi T (Low intensity)** |  |  |  |  |  |  | 12-16 reps (unspecified sets) | 55-65% 1RM |  |  |  |
| **Buchner DM** | 1997 | USA | 24-26 | 3 | Resistance machines | 8 | 2 sets 10 reps | 50-75% 1 RM | Unspecified | Yes | No training |
| **Thomas** | 1999 | USA | 16 | 4 | Resistance machines | 6 | Unspecified | 60% 1RM | Retest of 1RM every 4 weeks | 75% of sessions supervised | No exercise |
| **Hagerman FC** | 2000 | USA | 16 | 2 | Unspecified | 3 | 3 sets to failure (aim 6-8) | 85-90% 1RM | Unspecified | Yes | No training |
| **Hunter GR** | 2001 | USA | 25 | 3 | Resistance machines | 10 | 2 sets 10 reps | 80% 1RM | Retesting 1RM every 25 days | Yes | No training |
| **Cavani V** | 2002 | USA | 6 | 3 | Resistance machines | 9 | 1 set 12-15 reps | 12RM | Increased when >15 completed | Yes | No training |
| **Vincent** | 2002 | USA | 24 | 3 | Resistance machines | 13 | 1 set 8 reps | 80% 1RM | Adjusted according to RPE | Yes | No training |
| **Okazaki** | 2002 | Japan | 18 | 3 | Resistance machines | 5 | 2-3 sets 8 reps | 60-80% 1RM | Unspecified | Yes | No training |
| **Kallinen** | 2002 | Finland | 18 | 3 | Resistance machines | Unspecified | 3-4 sets 8-10 reps | 60-75% 1RM | 1RM retest 2 weeks | Yes | No training |
| **Haykowsky** | 2005 | Canada | 12 | 3 | Unspecified | Unspecified | 2 sets 10 reps | 50% 1RM, increased 2.5% weekly to 75% | Retest 1RM every 4 weeks | Yes | No training |
| **Madden** | 2006 | USA | 24 | 5 | Resistance machines + free weights | 10 | 3 sets 8-12 reps | 85% 1RM | 1Rm retesting every 4 weeks | Yes | No training |
| **Brentano** | 2008 | Brazil | 24 | 3 | Resistance machine + free-weight | 10 | 2-4 sets, 20-6 reps | 45-80% 1RM | 1RM retest every 8 weeks. | Yes | No exercise |
| **Lovell** | 2009 | Australia | 16 | 3 | Resistance machine | Lower extremities only | 3 sets 8 reps | 50-90% 1RM | 1RM retested every 4 weeks | Yes | No exercise |
| **Strasser** | 2009 | Austria | 24 | 3 | Unspecified | 8 | 3 sets 10-15 reps | 60-70% 1RM | Increase when >15 reps achieved. | Yes | No exercise |
| **Guido** | 2010 | Brazil | 24 | 3 | Unspecified | 9 | 1 set of 12, 10 and 8 reps | 60%, 70%, 80% 1RM | 1RM retest 4weeks | Unspecified | No exercise |
| **Martins** | 2010 | Portugal | 16 | 3 | Resistance band + bodyweight | 8 | 1 set of 8, increased to 3 sets of 15 | Unspecified | Unspecified | Yes | No training |
| **Bocalini** | 2010 | Brazil | 24 | 3 | Unspecified | 8 | 3 sets 10-12 reps | 60-70% 1RM | Adjusted 2-weekly | Yes | No training |
| **Kalapotharakos** | 2010 | Greece | 14 | 2 | Resistance machine | 6 | 3 sets 10 reps | 70% 3RM | 3RM retest every 2 weeks | Unspecified | No training |
| **Martins R** | 2011 | Portugal | 16 | Unspecified | Resistance band + bodyweight | 14 | 1 set of 12 reps, increased to 3 sets of 15 | Unspecified | Unspecified | Unspecified | No training |
| **Kanegusku** | 2011 | Brazil | 16 | 2 | Resistance machine | 7 | 2 sets 10 reps, progressed to 4 sets 6-8 sets | Progressed 70% 1RM to 85-90% 1RM | Unspecified | Yes | No exercise |
| **Locks** | 2012 | Brazil | 12 | 2 | Weighted shin pads | 7 | 3 sets 8 reps | 65-75% of 10RM | 10RM adjusted 3rd + 8th week | Unspecified | Health education every 4 weeks, no training |
| **Romero-Arenas S** | 2012 | Spain | 12 | 2 | Resistance machines | 6 | 3 sets of 12-6 reps | 50-100% of 6RM | 6RM adjusted by 2% if +/- 1 rep achieved | Yes | No training |
| **Wanderley** | 2013 | Portugal | 32 | 3 | Resistance machines | 9 | 2 sets 12-15reps | 50% 1RM, increased to 80% | 1RM adjusted 8 weeks | Yes | No training |
| **Schmidt** | 2014 | Denmark | 52 | 2/week for 12 weeks, then 3/week | Resistance machines + free weights | 5 | 3 sets increased to 4 sets, 16-20 reps, 12 reps, 10 reps, 8 reps. | Aim for failure at each set target rep | Unspecified | Yes | No training |
| **Anderson** | 2014 | Denmark | 16 | 2/week for 12 weeks, then 3/week | Resistance machines + free weights | 5 | 3 sets, 16-20 reps, 12 reps, 10 reps | Aim for failure at each set target rep | Unspecified | Yes | No training |
| **Lin SF** | 2015 | Taiwan | 16 | 3 | Resistance band | 10 | 2 sets of 4, increasing to 2 sets of 10 | Aim RPE 12-14 | Progress according to RPE | Yes | No training |
| **Irving BA** | 2015 | USA | 8 | 4 | Unspecified | Unspecified | 4 sets 8-10 reps | Unspecified | Training volume increase over initial 3 weeks | Yes | No training |
| **Oesen** | 2015 | Austria | 24 | 2 | Resistance band | 1-2 exercises for each 6-main muscle groups | 1-2 sets of 15 reps | Unspecified | Increase according to RPE | Yes | Cognitive training (no exercise training) |
| **Motalebi** | 2018 | Iran | 12 | 2 | Resistance band | 9 | 3 sets of 8-10reps | Using RPE (5-6 on OMNI scale) | Progress after 3sets of 8 achieved without fatigue | Yes | No training |
| **Costa** | 2018 | Brazil | 10 | 2 | Water based resistance training | 4 | 4 sets of 20 seconds, progressed to 8 sets of 10 seconds | Unspecified | Adjustment of rest period | Yes | Sham water based training |
| **Gargallo (High intensity)** | 2018 | Spain | 16 | 2 | Resistance band | 6 | 3-4 sets of 6 | 85% 1RM | Adjusted weekly according to RPE | Yes | No exercise |
| **Gargallo (Low intensity)** |  |  |  |  |  |  | 3-4 sets of 15 | 70% 1RM |  |  |  |
| **Zhong** | 2020 | China | 12 | Unspecified | Resistance band | 8 | Unspecified | Unspecified | Increase difficulty week 4 + 8 | Unspecified | No exercise |
| **Van Dongon** | 2020 | Holland | 12 | 2 | Resistance machine | 5 | 3-4 sets of 15 reps, increased to 4 sets of 8-12 | 50% 1RM increased to 75-80% | Unspecified | Yes | No exercise |
| **Timon** | 2021 | Spain | 24 | 3 | Resistance band + free weights | 9 | 3 sets 12-15 | Aim RPE 6-8 | Adjusted according to RPE | Yes | No exercise |
| **Elsangedy** | 2021 | Brazil | 12 | 3 | Resistance machine + free-weight | 8 | 3 sets of 15 reps | Participant selected weight | Participants adjusted weight | Yes | Board games |

**Appendix 2A: Study information, including study location, length, RET design, control design**

| **Reference** | **Number in Exercise** | **Number in control** | **Average age - Intervention (S.D.)** | **Average age - Control (S.D)** | **Average weight - Intervention (S.D.)** | **Average Weight - Control (S.D)** | **Male % Intervention** | **Male % control** | **Drop out Intervention** | **Drop out control** |
| --- | --- | --- | --- | --- | --- | --- | --- | --- | --- | --- |
| **Hagberg JM** | 23 | 13 | 72 (3) | | 74.5 (13.6) | 64.9 (14.5) |  |  | 4 | 1 |
| **Ades** | 12 | 12 | 69.9 (4) | 70.7 (5) | 75.6 (11.1) | 73.8 (10.8) | 50% | 41.70% |  |  |
| **Tsutsumi T (High intensity)** | 14 | 14 | 67.8 (4.9) | 69.8 (4.6) | 61.0 (9.6) | 60.9 (16.2) | 78.60% | 78.60% | 1 | 0 |
| **Tsutsumi T (Low intensity)** | 14 |  | 68.9 (7.6) |  | 65.9 (12.7) |  | 78.60% |  | 0 | 0 |
| **Buchner DM** | 25 | 30 | 74 | 75 |  |  | 48% | 50.00% | 6 | 1 |
| **Thomas** | 3 | 7 | 67 (1.73) | 68 (2.64) | 91.5 (4.5) | 84.1 (11.38) | 100 | 100 | x | x |
| **Hagerman FC** | 12 | 10 | 64.7 (5.0) | 66.2 (6.5) | 83.8 (17.6) | 80.2 (4.5) | 100% | 100.00% | 3 | 1 |
| **Hunter GR** | 14 | 8 | 67.3 (4.7) | 65.9 (4.0) | 75.0 (11.4) | 73.3 (24.1) | 57.10% | 50% | 0 | 0 |
| **Cavani V** | 22 | 15 | 69 (1.0) | 70 (4) |  |  | 36% | 40.00% | 0 | 0 |
| **Vincent** | 22 | 16 | 66.6 (6.7) | 71.0 (4.7) | 74.1 (14.8) | 73.1 (13.8) | 100% | 100.00% | Of original 84 recruited, 22 drop out. | |
| **Okazaki** | 8 | 7 | 64 (2.83) | 65 (5.29) | 61.6 (3.39) | 59.2 (9.52) | 100% | 100.00% |  |  |
| **Kallinen** | 16 | 11 | All aged 76-78 | | 66.9 (2.7) | 67.6 (3.9) | 0% | 0.00% | 4 | 0 |
| **Haykowsky** | 8 | 8 | 70 (4) | 67 (4) | 72.6 (18.4) | 75.3 (12.2) | 0% | 0.00% | 1 | 0 |
| **Madden** | 15 | 15 | 69.8 (5.81) | 71.8 (4.56) | 72.8 (19.36) | 74.2 (12.00) | 0% | 0.00% |  |  |
| **Brentano** | 10 | 9 | " | | 56.7 (5.8) | 61.4 (5.9) | 0 | 0 |  |  |
| **Lovell** | 12 | 12 | 74.1 (2.7) | 73.5 (3.3) | 79.4 (14.2) | 78.9 (11.3) | 100 | 100 | 0 | 0 |
| **Strasser** | 15 | 14 | 74 (5) | 74 (5) | 77.20 (11.51) | 66.29 (10.89) | 23.8 | |  |  |
| **Guido** | 25 | 25 | 68.04 (6.78) | 68.00 (6.38) | 60.96 (8.08) | 65.40 (13.06) | 0 | 0 |  |  |
| **Martins** | 14 | 13 | 73.2 (6.5) | 81.2 (7.9) | 74.1 (9.1) | 68.0 (13.6) | 35.7 | 46.20% |  |  |
| **Lobo AS** | 18 | 66 | 75.1 (8.3) | 77.6 (6.9) |  |  |  |  | 8 | 9 |
| **Bocalini** | 13 | 12 | 66 (9) | 64 (8) | 67.9 (1.3) | 69.1 (2.2) | 0% | 0 |  |  |
| **Kalapotharakos** | 7 | 7 | 83.4 (2.8) | 82.5 (3) | 81.7 (7.6) | 74.2 (7) | 100 | 100 |  |  |
| **Martins R** | 23 | 31 | 73.4 (6.4) | 77.7 (8.8) | 73.5 (8.0) | 70.9 (12.3) | 43.5 | 41.9 | 0 |  |
| **Kanegusku** | 13 | 11 | 63.1 (1) | 63.1 (1) | 72.6 (4.8) | 69.2 (3.3) | 38.50% | 18.20% |  |  |
| **Locks** | 21 | 35 | 69 (5) | 66.6 (6) |  |  | 44.4 | | 8 | 22 |
| **Romero-Arenas S** | 16 | 10 | 64.8 (4.5) | 58.0 (5.0) |  |  |  |  | 2 | 3 |
| **Wanderley** | 27 | 31 | 67.3 (4.9) | 67.8 (5.5) |  |  | 36.3 | 21.1 | 16 | 12 |
| **Schmidt** | 9 | 8 | 69.1 (3.1) | 67.4 (2.7) | 85.8 (12) | 89.3 (12.4) | 100 | 100 | 0 | 0 |
| **Anderson** | 9 | 8 | 69.1 (3.1) | 67.4 (2.7) | 85.8 (12) | 89.3 (12.4) | 100 | 100 | 0 | 0 |
| **Lin SF** | 54 | 54 | 73.9 (7.16) | 74.2 (7.04) |  |  | 37.5 | 34 | 6 | 7 |
| **Irving et al** | 10 | 10 | 70 (1) | 71 (2) | 75 (5) | 80 (5) | 50 | 50 |  |  |
| **Oesen** | 41 | 40 | 83.0 (5.5) | 83.4 ( 5.6) | 72.5 (9.7) | 73.0 (15.2) | 9.8 | 12.5 | 10 | 14 |
| **Motalebi** | 21 | 24 | 70.7 (6.6) | |  |  | 52.4 | 62.5 |  | x |
| **Costa** | 23 | 23 | 66.78 | 64.63 | 71.51 | 76.91 | 0 | 0 | 0 | 4 |
| **Gargallo (High)** | 39 | 23 | 71.10 (5.30) | 70.46 (8.1) | 64.67 (10.12) | 66.73 (8.73) | 0 | 0 | 3 | 2 |
| **Gargallo (Moderate)** | 31 |  | 68.74 (6.05) |  | 67.35 (10.68) |  | 0 |  | 3 |  |
| **Zhong** | 57 | 56 | 71.32 (1.09) | 71.26 (1.16) | 68.12 (1.19) | 68.56 (1.25) | 100 | 100 |  |  |
| **Van Dongon** | 82 | 86 | 75.7 (5.8) | 75.9 (6.5) | 76.3 (14.4) | 75.6 (13.6) | 37.8 | 40.7 | 10 |  |
| **Timon** | 18 | 19 | 70.35 (3.3) | 70.55 (4.0) | 70.9 (11.5) | 66.1 (10.2) |  |  | 0 | 0 |
| **Elsangedy** | 16 | 16 | 65.7 (3.3) | 66.3 (2.8) |  |  | 0 | 0 | 0 | 0 |

**Appendix 2B: Study demographics (Mean (S.D.))**

Appendix 3:


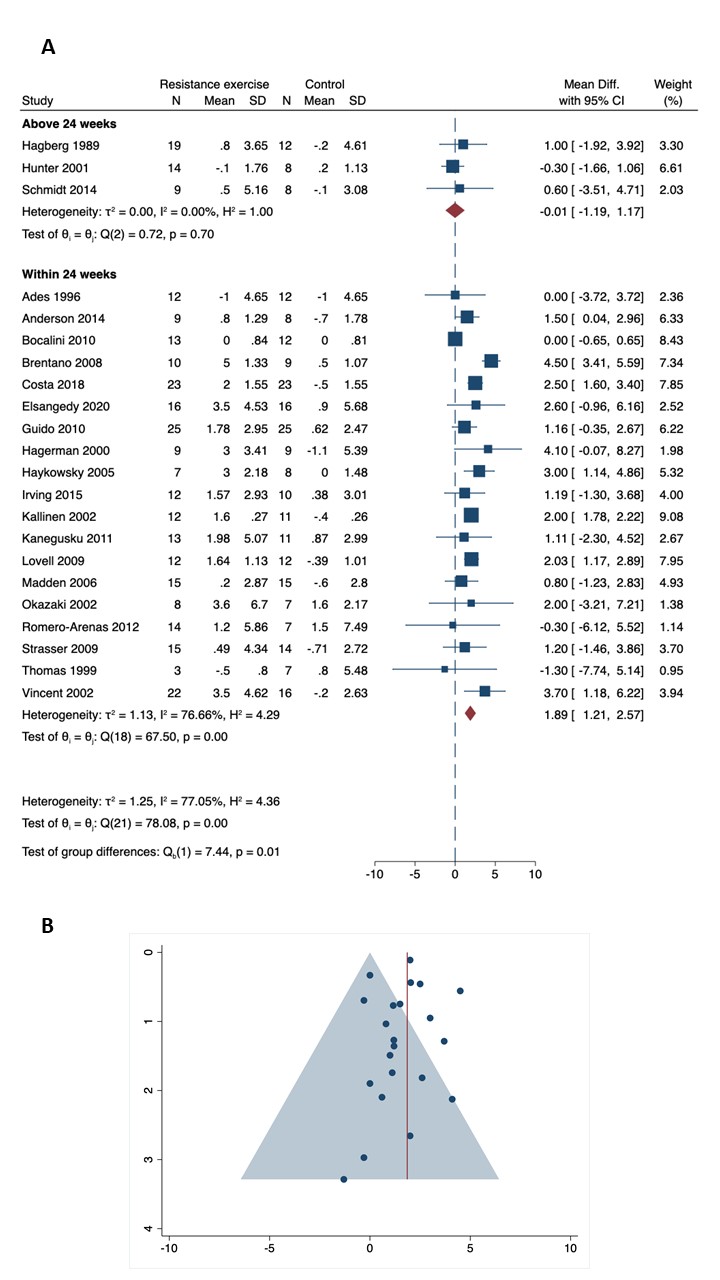


**Appendix 3A:** Contour-enhanced funnel plots of VO_2_ peak (p=0.54)


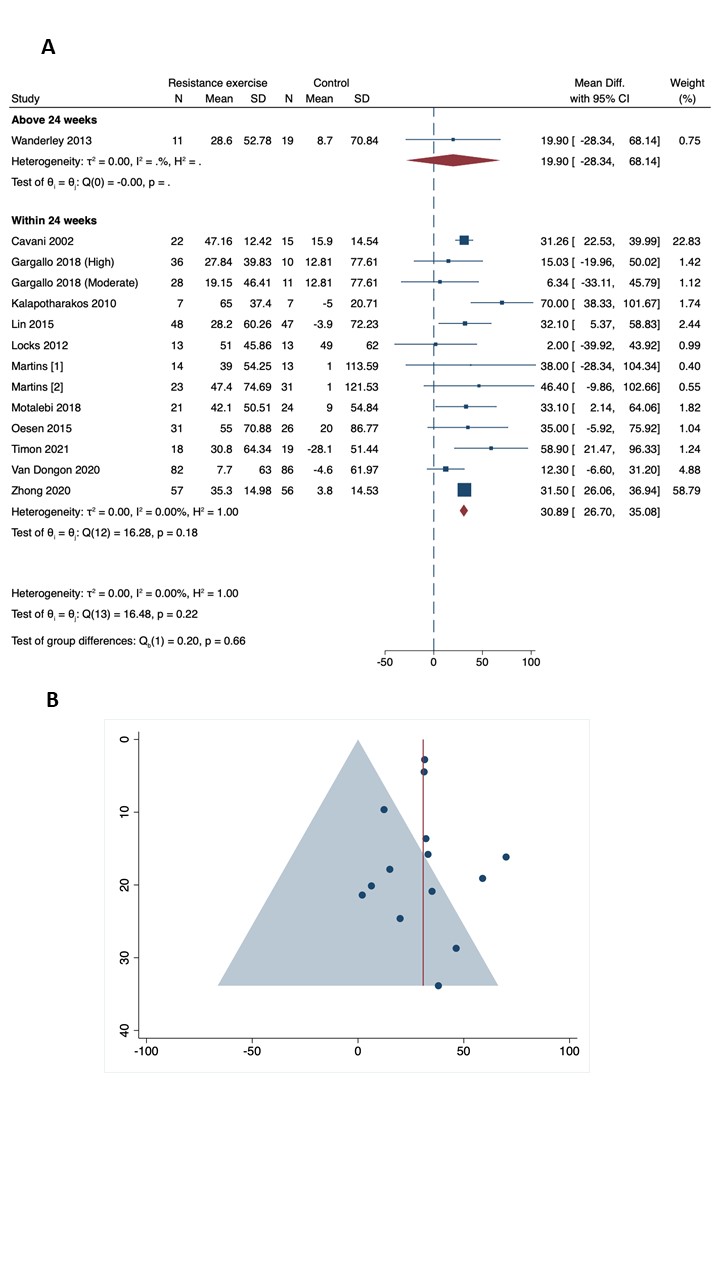


**Appendix 3B:** Contour-enhanced funnel plots of 6MWT (p=0.91)
